# Insights into the defensive roles of lncRNAs during *Mycoplasma pneumoniae* infection

**DOI:** 10.3389/fmicb.2024.1330660

**Published:** 2024-03-22

**Authors:** Zhujun Yang, Junjun Zhou, Nana Su, Zifan Zhang, Jiaxin Chen, Peng Liu, Peng Ling

**Affiliations:** ^1^Department of Critical Care Medicine, The Central Hospital of Shaoyang City and Affiliated Shaoyang Hospital, Hengyang Medical College, University of South China, Shaoyang, China; ^2^Institute of Pathogenic Biology, Basic Medical School, Hengyang Medical School, University of South China, Hunan Provincial Key Laboratory for Special Pathogens Prevention and Control, Hengyang, China

**Keywords:** lncRNAs, *Mycoplasma pneumoniae*, *Mycoplasma pneumoniae* infection, lncRNAs functions, regulatory mechanism of lncRNAs

## Abstract

*Mycoplasma pneumoniae* causes respiratory tract infections, affecting both children and adults, with varying degrees of severity ranging from mild to life-threatening. In recent years, a new class of regulatory RNAs called long non-coding RNAs (lncRNAs) has been discovered to play crucial roles in regulating gene expression in the host. Research on lncRNAs has greatly expanded our understanding of cellular functions involving RNAs, and it has significantly increased the range of functions of lncRNAs. In lung cancer, transcripts associated with lncRNAs have been identified as regulators of airway and lung inflammation in a process involving protein complexes. An excessive immune response and antibacterial immunity are closely linked to the pathogenesis of *M. pneumoniae*. The relationship between lncRNAs and *M. pneumoniae* infection largely involves lncRNAs that participate in antibacterial immunity. This comprehensive review aimed to examine the dysregulation of lncRNAs during *M. pneumoniae* infection, highlighting the latest advancements in our understanding of the biological functions and molecular mechanisms of lncRNAs in the context of *M. pneumoniae* infection and indicating avenues for investigating lncRNAs-related therapeutic targets.

## 1 Introduction

*Mycoplasma pneumoniae*, an atypical bacterium, is one of the smallest prokaryotic microorganisms without a cell wall (Shimizu, 2015). There are 200 known mycoplasma species, including six main species, which can cause human respiratory and reproductive tract diseases, among other diseases (Combaz-Söhnchen and Kuhn, 2017; Gómez Rufo et al., 2021). *M. pneumoniae* is one of the main pathogenic mycoplasmas, and it is a significant cause of respiratory tract infections. It causes endemic and epidemic primary atypical pneumonia, tracheobronchitis, pharyngitis, and asthma worldwide. *M. pneumoniae* pneumonia is the most significant disease associated with *M. pneumoniae* infection (Shimizu, 2016; Waites et al., 2017; Tsai et al., 2021). In addition, *M. pneumoniae* can cause infections outside the lungs (de Groot et al., 2017) by penetrating host cell membranes and invading respiratory tract mucous membranes, resulting in a pronounced inflammatory response outside the respiratory system (Poddighe, 2018). The severity of the diseases caused by *M. pneumoniae* ranges from mild to life-threatening (Waites et al., 2017). The dominant pathogenic mechanisms of *M. pneumoniae* are direct cytotoxicity and adhesion to host cells, immune evasion, and inflammation-induced damage (Jiang et al., 2021). The pathogenic mechanisms of extrapulmonary manifestations also involve direct injury mediated by inflammatory factors, indirect injury caused by the host immune response, and vascular occlusion (Hu et al., 2022).

Genes, which direct an organism’s development and function, include sequences with and without protein-coding functions (García-Andrade et al., 2022). Long non-coding RNAs (lncRNAs) comprise >200 nucleotides that do not code for proteins (Mattick et al., 2023). LncRNAs are widely expressed and play key roles in gene expression regulation. LncRNAs mainly interact with microRNAs (miRNAs), mRNAs, DNAs, and proteins, and they can thereby modulate gene expression in a variety of ways, e.g., by modulating chromatin function or regulating membraneless nuclear body assembly and function (Zhang et al., 2019; Statello et al., 2021a). LncRNAs are newly discovered regulators in many diseases, and there is a growing body of literature suggesting a relationship between lncRNAs and *M. pneumoniae* infection (Gu et al., 2020; Sun et al., 2022).

LncRNAs can be used by the host to modulate immune-related gene expression in order to resist *M. pneumoniae* invasion or decrease the damage caused by *M. pneumoniae* invasion, and *M. pneumoniae* can evade immune clearance by modulating the host lncRNAs (Wen et al., 2020).

This review summarizes the broad categories and common regulatory mechanisms of lncRNAs, the roles of lncRNAs in various diseases, and the defense mechanisms involving host cells’ lncRNAs against *M. pneumoniae* infection. It also provides an overview that indicates avenues for investigating lncRNAs-related therapeutic targets in *M. pneumoniae* infection and other diseases.

## 2 Category of lncRNAs

LncRNAs encompass a wide range of transcripts (Djebali et al., 2012) that exhibit significant diversity in terms of the presence of initiation codons, genomic location, and functional roles, making it difficult to easily characterize them. They can be broadly categorized into three types based on their mechanisms of action: (1) transcriptional regulation, (2) post-transcriptional regulation, and (3) other (Table 1) (Ma et al., 2013). The mechanisms of action of lncRNAs involved in transcriptional regulation can be further classified as (i) transcriptional interference, (ii) chromatin remodeling, and (iii) regulation effect. (The latter involves eRNAs, ncRNA-a1, Evf-2RNA, and Alpha-250/Alpha-280) (Table 1) (Ma et al., 2013). The mechanisms of action of lncRNAs involved in post-transcriptional regulation can be divided into (i) splicing regulation, (ii) translational control, lncRNAs that participate in translational control may function through binding to translation factors citation or ribosome (Ma et al., 2013), and (iii) other (the latter involves siRNA, 1/2-sbsRNA1, 21A, linc-MD1, IPS1, HULC, and BACE1-AS) (Table 1) (Rintala-Maki and Sutherland, 2009). The remaining lncRNAs can be classified into five categories based on other regulatory mechanisms: (i) protein localization (Watanabe and Yamamoto, 1994), (ii) telomere replication (Feng et al., 1995), (iii) RNA interference (Hellwig and Bass, 2008; Smekalova et al., 2016), (iv) regulation beyond transcription; unlike many other lncRNAs, promoter antisense RNAs (PAS RNAs) were initially considered to be merely passive transcription by-products of active promoters (Yang, 2022), and (v) translation regulation (Table 1).

**Table 1:** Conventional functions of lncRNAs.

Function of lncRNAs		lncRNAs	Reference
Transcriptional regulation	Transcriptional interference	DHFR upstream transcripts, SRG1 RNAs, 7SK snRNA, B2 SINE RNA, chromatin remodeling	Ma et al. (2013)
	Chromatin remodeling	*fbp1*, promoter RNAs, *Xist*, *MEG3*, *GAL10*-ncRNA, *HOTAIR*, *HOTTIP*, *COLDAIR*	
	Regulation effect	eRNAs, ncRNA-a1, Evf-2RNA, Alpha-250/Alpha-280	
Post-transcriptional regulation	Splicing regulation	MIAT, Malat 1, LUST	Rintala-Maki and Sutherland (2009) and Ma et al. (2013)
	Translational control	BC1, BC200, snaR, Gadd7, Zeb2, Zeb2NAT	
	Other	siRNA, 1/2-sbsRNA1, 21A, linc-MD1, IPS1, HULC, BACE1-AS	
Other	Protein localization	MeiRNA, ENOD40 RNA	Watanabe and Yamamoto (1994)
	Telomere replication	TERC	Feng et al. (1995)
	RNA interference	shRNAs or sgRNAs	Hellwig and Bass (2008) and Smekalova et al. (2016)
	Beyond transcription	PAS RNA	Yang (2022)
	Translation regulation	rncs-1	Ma et al. (2013)

## 3 Conventional lncRNAs regulatory mechanism

LncRNAs were initially thought to be merely interfering factors in gene transcription, (i.e., acting as accessory products that impede gene transcription involving RNA polymerase II), but they were later found to play essential roles in various biological activities. Notably, lncRNAs participate in transcription but prevent transcription by other chromosomes (Cabili et al., 2015).

The lncRNA LINC02159 (which is highly expressed in non-small cell lung cancer) forms a complex with Aly/REF export factor (ALYREF) through its 5-methylcytosine m^5^C modified sites and then binds to YAP1 mRNA, thereby increasing its stability (Chen et al., 2023). The lncRNA ADPGK-AS1, which mainly exists in mitochondria, is upregulated in artificially induced human M2 macrophages, and it binds to mitochondrial ribosomal protein MRPL35 and thereby promotes the tricarboxylic acid cycle and mitochondrial division, resulting in tumor growth (Karger et al., 2023).

**Figure 1:**
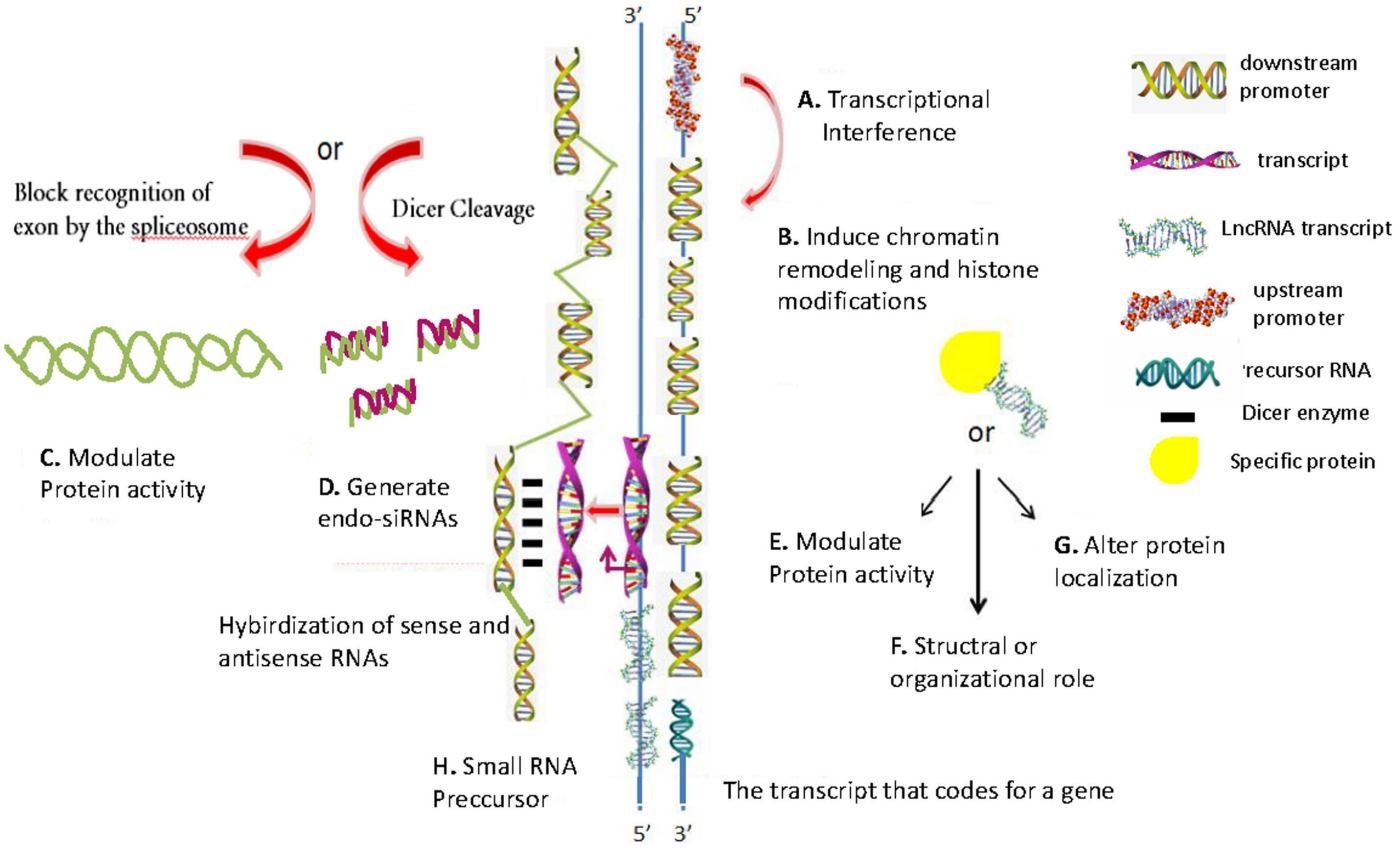
Schematic of conventional lncRNA regulatory mechanisms. **(A)** Transcription interference involves transcription from the upstream promoter region of a target protein-coding gene. **(B)** Inhibiting RNA polymerase II or inducing chromatin remodeling or histone modification, which interferes with target gene transcription. **(C)** Modulate protein activity. **(D)** Generating endogenous short interfering RNAs (endo-siRNAs), which target specific mRNAs (RNA interference). **(E)** Binding to a specific protein to modulate its activity. **(F)** Structural or organizational role, which catalyzes specific reactions. **(G)** Binding to a specific protein to alter its cellular localization. **(H)** Producing small RNA precursors. The diagrams are original and free of copyright restrictions.

The lncRNA MALAT1, also known as non-coding nuclear-enriched abundant transcript 2 (NEAT2), epigenetically regulates gene expression. Highly efficient knockdown of MALAT1 (using zinc finger nuclease-based technology) in extensive organization tumor cells confirmed that MALAT1 promotes *in vitro* and *in vivo* metastasis without affecting tumor cell proliferation (Gutschner et al., 2013). During extensive tumor cell proliferation, MALAT1 is regulated by multiple signaling pathways and has important roles in invasion and metastasis (Chen et al., 2022). MALAT1 regulates the activity of serine/arginine (SR) splicing factors, thereby influencing gene expression via alternative splicing (Tripathi et al., 2010). MALAT1 is also involved in cell cycle regulation, interacting with and promoting the cytoplasmic transport of heterogeneous nuclear ribonucleoprotein C (hnRNP C) in the G2/M phase, thereby controlling gene expression (Yang et al., 2011). Seven novel lncRNAs have been identified as competitive endogenous RNAs. Their abnormal expression leads to the widespread expression of tumorigenic genes (Figure 1A).

**Figure 2:**
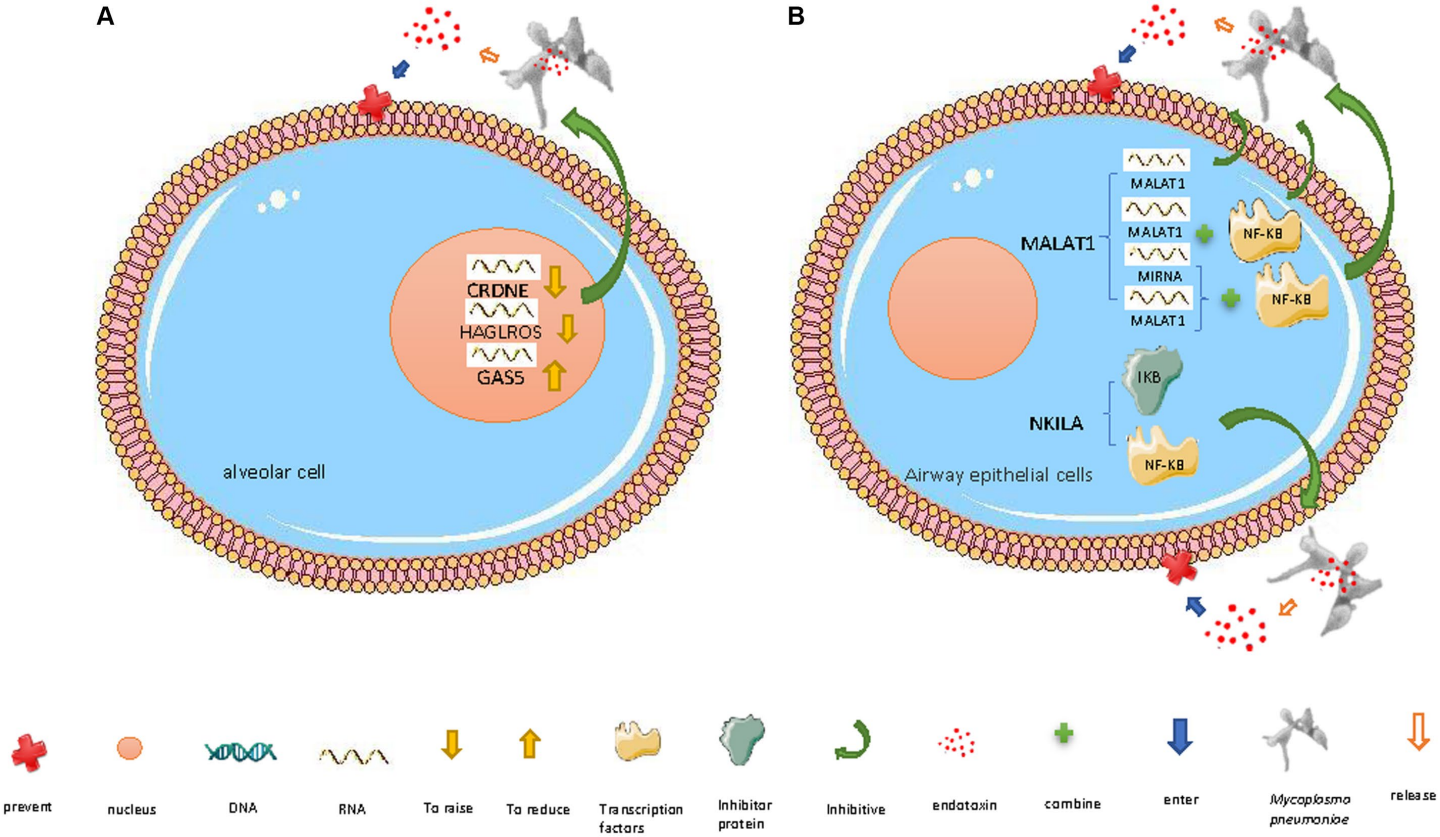
Mechanisms of lncRNAs defending against *M. pneumoniae* invasion. **(A)** In the nucleus: (i) lncRNA CRNDE downregulation, (ii) lncRNA HAGLROS downregulation, and (iii) lncRNA GAS5 upregulation inhibit *M. pneumoniae*-induced inflammatory factor release, thereby preventing damage to host cells. **(B)** In the cytoplasm: (i) downregulated lncRNA MALAT1 inhibits the inflammatory response triggered by *M. pneumoniae* endotoxin release by altering NF-κB activation; MALAT1 regulates NF-κB via three pathways: (a) competing with miRNAs that target NF-κB mRNA, (b) binding to NF-κB, and (c) directly resistance to endotoxins (ii) lncRNA NKILA inhibits the inflammatory response triggered by *M. pneumoniae* endotoxin release by preventing the dissociation of NF-κB from its inhibitor I-κB and thereby preventing NF-κB translocation to the nucleus. The diagrams are original and free of copyright restrictions.

As shown in the schematic in Figure 1, there are eight conventional lncRNAs regulatory mechanisms: (1) transcription interference involving transcription from the upstream promoter region of a target protein-coding gene (Figure 1A); (2) inhibiting RNA polymerase II or inducing chromatin remodeling or histone modification, which interferes with target gene transcription (Figure 1B); (3) generating complementary double strands involving mRNAs, which interferes with mRNA cleavage (Figure 1C); (4) generating endogenous short interfering RNAs (endo-siRNAs), which target specific mRNAs (RNA interference) (Figure 1D); (5) binding to a specific protein to modulate its activity (Figure 1E); (6) forming a ribozyme-protein complex, which catalyzes specific reactions (Figure 1F); (7) binding to a specific protein to alter its cellular localization (Figure 1G); and (8) producing small RNA precursors (Figure 1H).

Some upregulated lncRNAs play a tumor-promoting role, while downregulated lncRNAs in gastric cancer play a tumor-inhibitory role (Figure 1B) (Ahmed Shehata et al., 2021). Some lncRNAs can regulate protein activity (Figure 1C). SiRNAs or overexpression plasmids were transfected (with adequate transfection efficiency) into cells and verified using fluorescent markers (Figure 1D) (Cao et al., 2019). Some lncRNAs can form a complementary double strand with mRNA (which interferes with mRNA cleavage), and some lncRNAs can produce endo-siRNAs under the action of the Dicer enzyme (Figure 1E). Many lncRNAs are characteristically expressed in polarized tissues and specific cancer types (Xing et al., 2021). They form nucleic acid protein complexes with the proteins acting as structural components (Figure 1F) (Zhou et al., 2016), thereby altering protein localization (Figure 1G). LncRNAs (which are >200 nucleotides in length) have no protein-coding potential (Figure 1H).

## 4 LncRNAs in *M. pneumoniae* infection

### 4.1 LncRNAs in intrapulmonary *M. pneumoniae* manifestations

LncRNAs have the function of modifying cell biology (Statello et al., 2021b). LncRNAs can act with mRNAs, DNAs, proteins, and miRNAs to adjust gene expression at the epigenetic, transcriptional, post-transcriptional, translational, and post-translational levels in a variety of ways (Zhang et al., 2019). LncRNAs have many functions, including in *M. pneumoniae* infection, involving both: (1) transcriptional regulation, (2) post-transcriptional regulation, and (3) others (Table 1) (Wright et al., 2013). The interaction of these three regulatory mechanisms plays an important role in the *M. pneumoniae* infection of host cells (Dykes and Emanueli, 2017).

#### 4.1.1 Acute respiratory distress syndrome

LncRNAs are key regulators in respiratory diseases, and they can modulate cell growth arrest. The lncRNA GAS5 plays a significant role in many inflammatory diseases, including acute lung injury, idiopathic pulmonary fibrosis, and *M. pneumoniae* infection (Yang et al., 2021). GAS5 overexpression enhances cellular energy production and downregulates the pro-inflammatory cytokines IL-1β and IL-6 in human acute monocytic leukemia THP-1 cells (Figure 2A). The overexpression of miR-222-3p, which targets and reverses *M. pneumoniae-*induced THP-1 cell energy production, reduces *M. pneumoniae*-induced THP-1 cell viability, and accelerates the inflammatory response. GAS5 silencing reduces *M. pneumoniae*-induced chondrocyte activity and exacerbates *M. pneumoniae-i*nduced host cell inflammatory injuries. These findings offer new targets for treating *M. pneumoniae* infection (Yang et al., 2021). When exposed to host cells, *M. pneumoniae* upregulates the community-acquired respiratory distress syndrome acute respiratory distress syndrome (ARDS) toxin protein (encoded by the MPN372 gene), which is involved in host-cell interactions (Medina et al., 2012).

GAS5 was downregulated in lung epithelial cells treated with lipopolysaccharide (which can cause ARDS), suggesting that GAS5 is involved in the development of ARDS. The GAS5/miR-200c-3p/ACE2 signaling axis is involved in the apoptosis of ARDS lung epithelial cells. These findings offer new therapeutic targets for ARDS and enrich our understanding of the GAS5-mediated regulation of lung injury, which is of great significance for understanding the pathogenesis of ARDS (Li et al., 2018).

#### 4.1.2 Acute pneumonia

*M. pneumoniae* lipopolysaccharide can enter human embryonic lung WI-38 fibroblasts, induce inflammatory damage, and destroy the cells by triggering lncRNA HAGLROS upregulation. *M. pneumoniae* can induce inflammatory damage in WI-38 cells by modulating the miR-100/NF-κB axis. HAGLROS upregulation inhibits miRNA-100 (which therefore no longer targets and suppresses NF-κB3), thereby increasing NF-κB activity. HAGLROS knockout prevents NF-κB activation and thereby enhances WI-38 cell viability, inhibits apoptosis, and mitigates *M. pneumoniae*-induced cell damage (Figure 2A). Reducing the expression of miR-100 activates NF-κB3 and thereby causes WI-38 cell damage and apoptosis; this effect of reducing the expression of miR-100 can be prevented by NF-κB3 knockout (Figure 2A) (Liu et al., 2018). During *M. pneumoniae*-induced pneumonia, type I alveolar epithelial cells defend against *M. pneumoniae* infection by activating the innate immune response (Yamamoto et al., 2012), including the PI3K/AKT/NFκB pathway (Yang et al., 2021). The HAGLROS/miR-100/NF-κB axis may be a new target for the treatment of *M. pneumoniae* infection (Liu et al., 2018).

Another lncRNA that can regulate NF-κB activity is NKILA, which can exert an anti-inflammatory effect in airway epithelial cells. NKILA can mask the phosphorylation motif of I-κB (an inhibitor of NF-κB) and thereby prevent I-κB degradation and NF-κB translocation (Figure 2B) (Liu et al., 2015; Ke et al., 2018; Wang et al., 2018; Yu et al., 2018), inhibiting *M. pneumoniae*-induced inflammatory response genes (Peschke et al., 2014). NKILA is decreased and cytokines (IL-8 and TNF-α) are increased in bronchoalveolar lavage fluid from children infected with *M. pneumoniae* and NKILA knockdown in airway epithelial cells promotes *M. pneumoniae*-induced cytokine secretion NKILA exerts its anti-inflammatory effect by weakening the negative feedback loop of NF-κB signaling that regulates cytokine secretion (Figure 2B) (Zhang et al., 2021).

Moreover, downregulation of the lncRNA CRNDE and upregulation of miR-141 (which inhibits NF-κB and is targeted by CRNDE) inhibit the *M. pneumoniae* endotoxin-induced apoptosis and inflammatory response of human embryonic lung MRC-5 fibroblasts, thereby promoting cell survival (Figure 2A) (Zúñiga et al., 2012).

#### 4.1.3 Asthma

During *M. pneumoniae* infection, the host can regulate certain lncRNAs to inhibit inflammation and apoptosis. Activation of the transcription factor NF-κB, which regulates various inflammatory response genes, plays a significant role in *M. pneumoniae*-induced airway inflammation. Under normal conditions, NF-κB is bound to its inhibitor, I-κB, and remains in the cytoplasm. When stimulated by *M. pneumoniae* lipoproteins, which are recognized by toll-like receptors (TLRs), I-κB is phosphorylated and degraded, releasing NF-κB; activated phosphorylated NF-κB p65 then enters the nucleus and upregulates inflammation-related genes (Zhu et al., 2010). The pro-inflammatory mechanism of action of the lncRNA MALAT1 partially relies on it increasing NF-κB activation (by directly binding to it or indirect regulation, i.e., acting as a competitive endogenous RNA, competing with miRNAs that target NF-κB mRNA, and thus enhancing NF-κB activity). It thereby regulates the *M. pneumoniae*-induced inflammatory response (Figure 2B) (Dai et al., 2018; Lei et al., 2018). NF-κB upregulates TNF-α, which can damage capillary endothelial cells, thereby promoting microthrombosis and leading to ischemic necrosis, so TNF-α is associated with pneumonia severity (Figure 2B) (Salvatore et al., 2007). MALAT1 knockdown inhibits *M. pneumoniae*-induced NF-κB p65 phosphorylation in mouse airway epithelial cells and mouse lung tissue. Thus, the regulatory role of MALAT1 in *M. pneumoniae* infection-induced inflammation is closely related to NF-κB activation (Zhang et al., 2021).

The mechanism by which lower respiratory tract *M. pneumoniae* infections trigger or worsen asthma in children is not completely clear (Kumar et al., 2019). Following *M. pneumoniae* infection in children, a small percentage of individuals present with recurrent wheezing episodes, and the prevalence of *M. pneumoniae* infection in children with acute asthma has been reported to be 46% (Kassisse et al., 2018). *M. pneumoniae* can induce mucin overproduction by inhibiting the transcription suppressor FOXA2 Lung function is improved by restoring FOXA2’s transcription suppressor function and downregulating goblet cell hyperplasia and metaplasia (GCHM)-promoting pathways in *M. pneumoniae*-infected airways in asthma patients with abnormal mucin secretion and accumulation in airway lumens, which are clinical markers of asthma (Hao et al., 2014). In addition, asthma is associated with upregulated MALAT1 and downregulated miRNA-216a (which is inhibited by MALAT1, acting as a molecular sponge), while the opposite (MALAT1 downregulation and/or miRNA-216a upregulation) significantly increases apoptosis while significantly decreasing cell proliferation, migration, and invasion (Huang J. et al., 2021).

### 4.2 Immune-mediated mechanisms of *M. pneumoniae* extrapulmonary manifestations

*M. pneumoniae* can cause various extrapulmonary manifestations, including those that affect the cardiovascular system, skin, and liver.

The cardiovascular manifestations of *M. pneumoniae* infection (Bakshi et al., 2006) include aortic thrombosis (Flateau et al., 2013) and pulmonary thrombosis. *M. pneumoniae* can directly spread via the blood to distant organs and induce local production of cytokines and chemokines (TNF-α and IL-8), eventually leading to local vasculitis or thrombosis. *M. pneumoniae* can also indirectly lead to systemic hypercoagulability by activating chemical mediators, complement, and fibrin D-dimer, which increase the risk of thrombotic vascular occlusion (Hu et al., 2022).

The dermatological manifestations of *M. pneumoniae* infection include erythema nodosum [an immune complex-mediated disease that primarily affects young women (Kakourou et al., 2001)] and cutaneous leucocytic vasculitis [characterized by perivascular neutrophilia reported to be caused by circulating immune complexes (Kakourou et al., 2001; Perez and Montes, 2002)]. Although *M. pneumoniae* cannot infect the squamous cell epithelium, it may produce inflammatory bullous lesions due to the transfer of cytokines from the respiratory tract to the skin via the blood (Narita, 2016).

The hepatic manifestations of *M. pneumoniae* infection can arise as a result of modulation of T-cell-mediated immune responses by T cell immunoglobulin and mucin domain-containing proteins (TIMs) expressed on T cells, which can regulate T cell cytokine differentiation (Wang et al., 2008). Liver damage can also be caused by inflammatory cell activation induced by signaling involving TLR2 and TLR4, which are expressed on cell surfaces and can detect and initiate responses to extracellular pathogens (Kawasaki and Kawai, 2014; Shimizu et al., 2014). *M. pneumoniae* causes acute and severe hepatitis in children, which is likely to be immune-mediated and involve both innate and adaptive immune responses (Poddighe, 2020).

In summary, the detailed mechanisms underlying the three abovementioned types of *M. pneumoniae* extrapulmonary infection are unclear, but it is clear that they generally involve inflammatory immune responses (Poddighe et al., 2022).

### 4.3 LncRNAs and immune-mediated mechanisms of *M. pneumoniae* infection

#### 4.3.1 Intrapulmonary *M. pneumoniae* manifestation

*M. pneumoniae* adhesion molecules and metabolites can cause immune damage to respiratory epithelial cells. *M. pneumoniae* infection decreases CD4^+^T cell function, which is the primary cause of immune dysfunction in patients with *M. pneumoniae* infection, impairing antigen presentation, B-cell maturation, and antibody production. *M. pneumoniae* also disrupts other humoral and cellular immune responses (Hu et al., 2022). During host cells’ non-specific immune defense against *M. pneumoniae*, lncRNAs regulate reactive oxygen species production by NADPH oxidase to fight *M. pneumoniae* (Lee et al., 2020). LncRNAs can also be exploited by *M. pneumoniae* to evade the immune system (Hu et al., 2022).

#### 4.3.2 Extrapulmonary in children infected (neurological) *M. pneumoniae* manifestations

*M. pneumoniae* infection-induced neurological diseases are likely to be a result of immune responses to the infection, based on indirect immunofluorescence and PCR analysis of cerebrospinal fluid samples from patients with these neurological diseases (Poddighe, 2018).

The lncRNA NKILA was downregulated while IL-8 and TNF-α were upregulated in children infected with *M. pneumoniae*. NKILA knockdown *in vitro* promotes the inflammatory effect of *M. pneumoniae* on A549 and BEAS-2B respiratory epithelial cells (Zhang et al., 2021). IL-8 and TNF-α are two well-known pro-inflammatory cytokines that play crucial roles in airway inflammation and chemotaxis caused by *M. pneumoniae* (Martin et al., 1997).

### 4.4 LncRNAs/circRNAs in drug-resistant *M. pneumoniae* infection

Both macrolide-resistant and refractory *M. pneumoniae* infections complicate the clinical management of *M. pneumoniae* pneumonia (Tsai et al., 2021). Macrolide-resistant *M. pneumoniae* harbors a point mutation in 23S rRNA domain V (with substitutions mainly detected at positions 2063 and 2064) (Yang et al., 2017). Circular RNAs (circRNAs), which are like lncRNAs but form a closed loop (Ashekyan et al., 2022), play important roles in gene expression regulation by sequestering miRNA targets (acting as molecular sponges) (Meng et al., 2017). The miRNA targets of circRNAs (detected by high-throughput sequencing) could be utilized as biomarkers for the diagnosis of early-stage refractory *M. pneumoniae* pneumonia (Huang F. et al., 2021).

## 5 LncRNAs in other diseases

LncRNAs affect cardiovascular development, including the embryonic development of the heart and vascular system (Kohlmaier et al., 2023). The lncRNA CARMEN can regulate the fate, differentiation, and homeostasis of human cardiac progenitor cells (Ounzain et al., 2015). Additionally, lncRNAs serve as key regulators in cardiovascular diseases such as arterial hypertension, coronary heart disease, and acute myocardial infarction (Correia et al., 2021). For example, overexpression of lnc-Ang362 indirectly activates the (NF-κB) signaling pathway, which promotes vascular smooth muscle cell proliferation and migration, thereby aggravating arterial hypertension (Wang et al., 2020). Additionally, upregulation of the lncRNA cardiac hypertrophy-related factor (CHRF) in cardiomyocytes can upregulate myeloid differentiation primary response 88 (MYD88), inducing cardiomyocyte hypertrophy and apoptosis, leading to heart failure (Wang et al., 2014).

LncRNAs also regulate the development and differentiation of neurons and the nervous system, and they play various pathological roles, leading to various neurodegenerative diseases (Nadhan et al., 2022). In Alzheimer’s disease, the highly upregulated antisense lncRNA BACE1-AS stably binds to BACE1, enhancing the production of β-amyloid plaques (Zeng et al., 2019). In schizophrenia, the reduced expression of the lncRNA MIAT is associated with behavioral changes (Ip et al., 2016). In autism spectrum disorder, the lncRNA SYNGAP-AS1 can downregulate SYNGAP1, causing cortical functional impairment (Velmeshev et al., 2013). In ischemic stroke, the reduced expression of the lncRNA MEG3 activates the Notch signaling pathway and thereby promotes angiogenesis (Yan et al., 2016).

In cancer, some lncRNAs have been identified as oncogenes, while others have been identified as tumor suppressors (Nadhan et al., 2022). The lncRNA HOTTIP acts as an oncogene in acute myeloid leukemia, where it is abnormally elevated and functions as an epigenetic regulator, modulating hematopoietic gene-associated chromatin signatures and transcription (Luo et al., 2019). The p53-dependent lncRNA PVT1 inhibits lung cancer by downregulating c-Myc (Olivero et al., 2020). The abovementioned lncRNA CHRF plays a crucial role in the progression of various tumors, such as prostate cancer, by miRNA binding (Gai et al., 2019). The lncRNA LUCAT1 is associated with various cancers, including cervical cancer, where it exerts oncogenic functions by sequestering miR-181a (Xing et al., 2021). Finally, the highly expressed lncRNA NEAT1 sequesters miR-155 and upregulates TIM3, which promotes CD8 T cell apoptosis and thereby facilitates hepatocellular carcinoma immune evasion and development (Yan et al., 2019).

In endocrine diseases such as diabetes and related conditions (including diabetic nephropathy, diabetic retinopathy, and diabetic neuropathic pain), dysregulated lncRNAs have significant effects (Alipoor et al., 2021). For example, the downregulation of the lncRNA H19 disrupts mitochondrial fatty acid β-oxidation and leads to fatty acid accumulation and insulin resistance (Gui et al., 2020). The lncRNA PVT1 is overexpressed in diabetic nephropathy. PVT1 silences FOXA1 by directly binding to and stabilizing the histone methyltransferase EZH2 to induce trimethylation-based silencing (Liu D. W. et al., 2019). The reduced expression of FOXA1 induces podocyte apoptosis, contributing to the progression of diabetic nephropathy. The lncRNA MALAT1 sequesters miR-125b and thereby upregulates target genes, promotes neovascularization, and impairs vision (Liu P. et al., 2019). The lncRNA NONRATT021972 is upregulated in diabetic neuropathic pain, which it exacerbates by upregulating TNF-α and purinergic receptors (P2X) 3 and 7 It increases the expression of TNFα as well as purinergic receptors (P2X) 3 and 7 (Peng et al., 2017).

### 5.1 Small molecule response induced by lncRNAs in *M. pneumoniae* infection

Neutrophils are one of the cells that respond to inflammation sites and play a vital role in killing pathogens (Schenten et al., 2018). The inflammatory response caused by neutrophil activation can be triggered by endogenous ligands called damage-associated molecular patterns (DAMPs) or actively aerated alarmins (Chan et al., 2012). Recently, S100A8/9 proteins have been identified as DAMPs released by neutrophils and monocytes [which has been proposed to be an active process dependent on the microtubule network (Schiopu and Cotoi, 2013) or a process involving NETosis (Ehrchen et al., 2009; Bianchi et al., 2011)]. The elevation of S100A8/9 increases neutrophils in the blood, which can promote the occurrence of atherosclerotic disease due to neutrophil accumulations in artery walls (Schiopu and Cotoi, 2013).

## 6 LncRNAs as targets for treating *M. pneumoniae* infection

LncRNAs in the nucleus (e.g., CRNDE, HAGLROS, and GAS5) and cytoplasm (e.g., MALAT1 and NKILA) work together to resist *M. pneumoniae* invasion. Downregulation of CRNDE can upregulate miR-141 and thereby inhibit lipopolysaccharide-induced MRC-5 fibroblast apoptosis and the associated inflammatory response (Meng et al., 2019). HAGLROS downregulation ameliorates lipopolysaccharide-induced PI3K/AKT/NF-κB pathway activation and inflammatory damage in WI-38 cells by causing a lack of HAGLROS to compete with miRNA-100, leading to NF-κB3 downregulation (Torrealba et al., 2020). The HAGLROS/miR-100/NF-κB axis may provide a new target for the treatment of acute-phase *M. pneumoniae* pneumonia (Fang and Shi, 2022). Highly expressed lncRNA GAS5 reduces the inflammatory response and the viability of LAMP-1-induced human acute monocytic leukemia THP-1 cells by targeting the miR-222-3p/TIMP3 axis (Yang et al., 2021). Downregulated MALAT1 plays a key regulatory role in reducing *M. pneumoniae*-induced inflammation (Zhao et al., 2016) by downregulating NF-κB signaling (Shimizu et al., 2008). NKILA inhibits the *M. pneumoniae*-induced inflammatory response of airway epithelial cells by modulating NF-κB (Zhu et al., 2019).

The findings that lncRNAs/circRNAs carried by exosomes in breast cancer (BC) regulate breast cancer-related target genes (Ashekyan et al., 2022) prompt the question of whether the lncRNAs/circRNAs/target genes are related to *M. pneumoniae* infection and whether they may represent novel targets for the treatment of *M. pneumoniae* (Tang et al., 2020). LncRNAs have been shown to have broad clinical applications, including cancer diagnosis and prognosis biomarkers (Ashekyan et al., 2022).

## 7 Perspectives

Although recent lncRNA sequencing analyses have identified potentially key lncRNAs associated with *M. pneumoniae* pneumonia (Huang et al., 2016), their biological roles and function mechanisms remain largely unknown (Chen et al., 2018). It is important to determine the pivotal molecular mechanisms underlying *M. pneumoniae* pneumonia in order to develop effective treatment strategies (Chen et al., 2018). Studying lncRNAs may provide an academic foundation for more comprehensive understanding of the molecular mechanisms underlying *M. pneumoniae* pneumonia and for identifying effective treatment targets, thereby identifying unconventional strategies for the treatment of acute-phase *M. pneumoniae* pneumonia.

LncRNA regulates cardiovascular development (Correia et al., 2021) and the development and differentiation of neurons and the nervous system (Nadhan et al., 2022). In cancer, some lncRNAs have been identified as oncogenes, while others have been identified as tumor suppressors (Nadhan et al., 2022). In endocrine diseases such as diabetes and related conditions, dysregulated lncRNAs have significant effects (Alipoor et al., 2021). LncRNAs play a variety of roles in these diseases, which may provide insights into the currently unknown roles of lncRNAs in various *M. pneumoniae* infection states.

## 8 Conclusion

LncRNAs encompass a wide range of transcripts with significant diversity in terms of the presence of initiation codons, genomic location, and functional roles. They are newly discovered regulators in many diseases, and there is a growing body of literature suggesting a relationship between lncRNAs and *M. pneumoniae* infection. In this review, we broadly classified lncRNAs’ mechanisms of action as transcriptional regulation, post-transcriptional regulation, and others, and detailed the conventional mechanisms of action of lncRNAs. We also discussed lncRNAs’ roles in the pathogenesis of four major disease types (cardiovascular diseases, neurological disorders, cancers, and the endocrine disease diabetes). Furthermore, we provided insights into lncRNAs’ key protective roles against intrapulmonary, extrapulmonary, and drug-resistant *M. pneumoniae* infections. This review serves as a succinct overview and indicates avenues for investigating lncRNAs’ roles as novel therapeutic targets.

## Author contributions

ZY: Writing – original draft, Project administration. JZ: Writing – original draft. NS: Writing – original draft. ZZ: Writing – original draft. JC: Writing – original draft. PLiu: Writing – review & editing, Writing – original draft, Supervision, Resources. PLin: Supervision, Writing – review & editing.
